# Towards Reliable Prosody Detection in Teaching Practical Phonetics: A Sustainable Digital Approach

**DOI:** 10.12688/f1000research.178913.2

**Published:** 2026-07-10

**Authors:** Olesya Tolstykh, Alexey Svatov, Tamara Oshchepkova

**Affiliations:** 1 The Department of Modern Languages and Communication, National University of Science and Technology MISIS, Moscow, Russian Federation; 2 Liberal Arts Department, American University of the Middle East, Egaila, Kuwait, 54200, Kuwait

**Keywords:** automated prosody detection system, machine learning in phonetics, suprasegmental analysis, Teaching English as a Foreign Language (TEFL), convolutional neural networks (CNN), Label Studio annotation

## Abstract

This research focuses on providing quality education to specialists in the field of linguistic studies, phonology, theoretical and practical phonetics by creating an innovative automated service which can ease the linguists endeavour to transcribe and examine suprasegmental features of human speech prosody. The authors attempted to design a docker-based architecture for prosody labelling. The pre-processing stage compiled 47 manually marked recordings. The data-processing stage produced a 1.9 GB Parquet corpus of 882 denoised, labelled clips via *librosa*, *noisereduce*, and *Label Studio.* The model selection stage compared 4909 scikit-learn models and 120 CNNs. The strongest classical approach (Random Forest) reached 0.373 accuracy, whereas the best CNN scored 0.455. Having recognised the prototype limitations, the researchers have scheduled a roadmap for improving the tool. The suggested model applies the principles of machine learning to automatically generate prosodic analysis that extends beyond individual sound segments and reflects suprasegmental aspects of prosody including rhythm, intonation, and phrasal stress.

**Abbreviations**
AI: Artificial Intelligence
AMQP: Advanced Message Queuing Protocol
CEFR: Common European Framework of Reference
CNN: Convolutional Neural Networks
IPA: International Phonetic Alphabet
L1: First Language, Mother Tongue
L2: Second Language
LMS: Learning Management System
ML: Machine Learning
RP: Received Pronunciation
TEFL: Teaching English as a Foreign Language

## Introduction

Foreign language teaching despite being an integrative process is often subdivided into teaching language aspects such as teaching vocabulary and grammar, developing productive and receptive skills. Teaching phonetics is often treated as a minor subordinate strand, the main purpose of which is to uphold the development of foreign language skills. The rationale behind such a position might be partially explained by the fact that many educators misinterpret communicative approach and place excessive emphasis on teaching the ways to deliver the message by sacrificing accuracy of the utterances ( Levis, 2022; Low, 2021). As a result, teaching pronunciation has become a neglected and increasingly marginalized area. The core philosophy of the communicative approach, however, does not imply refusal from teaching pronunciation because the lack of attention to mastering pronunciation may result in less effective communication due to misinterpretations of spoken messages and poorer listening comprehension skills.

By integrating pronunciation instruction into language education, teachers promote more inclusive and equitable learning opportunities, ensuring that learners develop the communicative competence needed to participate fully in academic, professional, and social contexts. Clear pronunciation enhances confidence and employability, thereby supporting lifelong learning and social mobility, while also fostering cross-cultural understanding. In this way, teaching pronunciation contributes not only to individual empowerment but also to the broader sustainability agenda of reducing barriers to participation in an interconnected world.

Given the variety of existing accents, there is no agreement among educators about the model of intelligible articulation that should be selected for teaching. In the updated edition of Common European Framework of Reference (CEFR) the requirement of pronunciation of a native speaker as a standard has been replaced by the intelligibility criterion ( Council of Europe, 2020). This implies the ability to articulate phonemes and convey prosody, including intonation, rhythm and stress. Foreign language learners are expected to develop natural pronunciation and intonation that is comprehensible to interlocutors and recognise regional and sociolinguistic varieties of pronunciation. In other words, the mastery of pronunciation should be sufficient to achieve the needs for English as a lingua franca, which undermines the notion of native speakerism and promotes the concept of intelligibility ( Almusharraf, 2024; Jeong, & Lindemann, 2025). This approach remains a subject of debate because it is difficult to discern and measure what exactly intelligible accent constitutes ( Dillon, & Wells, 2023).

Although there has been a shift toward prioritizing intelligibility over standard pronunciation, a preference for a recognised language norm known as Received Pronunciation (RP) persists among educators and students ( Pitychoutis, 2024). Within the context of the current research, authors have also selected RP, the reasons for which are explained in the Methodology section.

Despite the fact that pronunciation is a significant part of language instruction, some teachers exclude it from their lessons ( Couper, 2021). In classes that address pronunciation, explicit instruction is relatively uncommon and is typically included as part of remedial work. Taking this a step further, teaching pronunciation as an autonomous subject or skill is even less frequent ( Pennington, 2021). Regarding the content of pronunciation-related classroom activities, they often emphasize the importance of correctness of segmentals like phonemes or word stress while many seem to be less concerned about suprasegmentals like word junctions or intonation, meaningful use of chunking and phrasing ( Bøhn, & Hansen, 2017; Couper, 2021; Foote, et al., 2016). Segmental features are believed to be “more teachable” ( Elnagar, 2020) while suprasegmentals are difficult to describe without reference to specialised terminology. Segmental errors are perceived as more salient and easier to correct. However, rhythm and intonation have special significance in accomplishing reasonable pronunciation with sentence stress accounting for almost 36% of the speaking variance ( Ma, et al., 2018). The context of this research requires explicit instruction with particular emphasis on suprasegmental language elements because mastering them is a requirement for the specialists in the field of linguistic studies, phonology, theoretical and practical phonetics ( Gordon, 2023; Stratton, 2023).

Most pronunciation teaching methods are rooted in the audio-lingual approach and are aimed at developing accuracy via repetition and imitation. The most frequently applied classroom techniques are listening to target sounds, controlled mechanical imitation, comparison of pronunciation models, choral repetition, explanation of the sound–spelling correspondence, and corrective feedback.

Modern technologies are widely used in foreign language education, with AI-powered tools gaining popularity and proving to be effective for second language (L2) pronunciation teaching. Almost 70% of the participants in the study conducted by Almusharraf (2024) reported using technology for teaching pronunciation. Namely, teachers are using ChatGPT for practicing, obtaining explanations and examples of L2 phonetic features ( Mompean, 2024). Among potent AI-powered applications for improving English pronunciation and speaking abilities experts name a few prominent speech recognition technologies including Speechling, Duolingo, and Google Assistant ( Dennis, 2024). AI-powered chatbots leverage voice recognition technology to simulate structured conversations with users and offer immediate feedback on their spoken input ( Sonsaat-Hegelheimer & Kurt, 2024). These developments are supported by machine learning, which has enabled automated scoring of learners’ performance, including pronunciation accuracy. For instance, Pearson Education Inc. (2022) claims that their scoring system can measure “the position and length of pauses, the stress and segmental forms of the words, and the pronunciation of the segments in the words within their lexical and phrasal context” (p. 19). The observations of some researchers ( Pennington, 2021), however, point to the limited value of the available technology for teaching and learning pronunciation, especially in the areas of suprasegmental features such as intonations, rhythm, and pitch. Besides, educators report multiple fallacies in error detection and feedback provided by automated speech recognition systems as well as issues of reliability and validity with automated scoring generated by AI ( Rogerson-Revell, 2021).

One of the applications that has been used by phoneticians for over two decades is Praat authored by Paul Boersma and David Weenink (2025) from the University of Amsterdam. It is a free software that allows users to automate routine activities of analysing acoustic parameters of speech ( Jadoul, et al., 2024). The core functionalities of Praat include detailed examination of pitch, formants, intensity, voice quality, and the production of spectrograms and cochleagrams. The programme also supports acoustic- and articulatory-based speech synthesis, signal manipulation (e.g., controlled changes in pitch, intensity, and duration), annotation based on International Phonetic Alphabet (IPA), and segmentation into words or phonemes. Advanced modules address Optimality Theory, neural-network modelling, and multivariate statistics ( Boersma, et al., 2020).

Interestingly, Praat has been extensively used in medical research as a tool helping patients with speech disorders ( Gölaç, et al., 2025; Karia, 2023; Sonkaya, et al. 2024). The number of empirical research papers that discuss the use of Praat for educational purposes is considerably lower. Still, those scientists who have analysed the impact of Praat-based language learning activities shared their observations about benefits and limitations of applying Pratt in an English-learning classroom. Thus, several researchers and practitioners have reported Praat as an effective tool for teaching various aspects of pronunciation. In particular, educators found useful the visual feedback provided by Praat. Automatically generated sound wave spectrogram and the pitch tone graph, like the ones presented in Figure 1, allow learners to compare their pitch contours to the provided samples, identifying discrepancies in intonation ( Guo, 2025; Larassati et al., 2022; Wang, 2021; Zeng, & Huang, 2025). This feature helps in overcoming the challenges that the abstract nature of pronunciation poses for language learners ( Topal, 2024).

**Figure 1.**
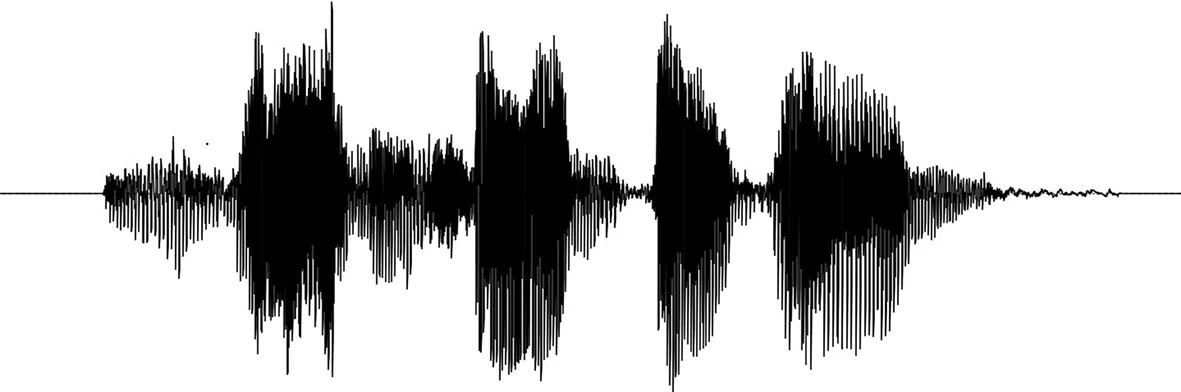
A sample of a spectrogram created by Praat.

Another benefit created by Praat is the opportunity for autonomous practice since students are empowered to self-monitor their pronunciation outside classrooms by recording and analyzing their speech ( Osatananda, & Thinchan, 2021). Therefore, empirical studies confirmed that students who were introduced to Praat as a tool for enhancing their pronunciation demonstrated significantly greater progress than the control group ( Chen, 2022; El-Garawany, 2021). Researchers believe that this software also helps teachers to analyse pronunciation difficulties that learners face and plan remedial work accordingly ( Wang, 2024).

Despite the stated aids provided by Praat, there are distinct limitations that restrict its wide use in the classroom. First of all, although the software is simple to operate and has intuitive graphics, understanding the generated spectograms requires some knowledge of acoustics and phonetics ( Rogerson-Revell, 2021; Yang, & Zhao, 2021). Besides, teaching students to interpret spectrograms might be time-consuming. If teachers decide to encourage learners to use Praat for self-study, they have to provide preliminary training in reading the visuals provided by the software.

While its specialised focus constitutes a major strength, Praat’s interface and architecture remain relatively archaic. Since Praat was first released in 1991, there have been multiple attempts to improve it not only on behalf of its developers but also users. For instance, de Jong, et al. (2021) endeavoured to create Praat script for measuring fluency by detecting filled pauses. Another group of researchers has been working on making Praat functionality available in Python which could provide alternative means of interaction with Praat’s algorithms ( Jadoul, et al., 2024). However, none of these attempts meet the needs of educators and linguists who intend to use Praat for deeper understanding of pronunciation phenomena.

To sum it up, there is clear evidence of extensive research being done in the area of teaching foreign language pronunciation. Scientists emphasize the importance of intelligible articulation for enhancing communication and mutual understanding. Instructions related to suprasegmental elements contribute significantly to producing meaningful discourse. Mastering pronunciation is significantly enhanced and eased if modern computer technologies and AI-powered tools are applied. However, none of the research projects that the authors reviewed analyse the specific settings of teaching pronunciation to the students who specialize in the field of linguistic studies, phonology, theoretical and practical phonetics. The requirement of intelligibility is not sufficient at this level. These learners are required to recognize different varieties of pronunciation, analyse acceptable and unacceptable deviations from the standard, back up their explanations with the theoretical justifications, and perform the role of a model of the pronunciation that is close to the language norm. Therefore, some specialized tools might be required apart from those that are already used for teaching pronunciation. The current research demonstrates an attempt to develop a tool that is suitable for the stated purposes.

## Research context and objectives

The current research is being implemented at the National University of Science and Technology (NUST “MISIS”) within the context of the course “Practical Phonetics of English”. The original syllabus for the course was developed by Professor PhD Sukhova N. V., with the academic support of her colleagues Professor PhD Tolstykh O. M. and Professor Gendelev I. D. This course is compulsory for the students who major in linguistics and specialize in translation, Teaching English as a Foreign Language (TEFL), or media and communication.

The primary objective of this study is to develop and describe a prototype system for automated prosody analysis that integrates contemporary speech-processing techniques and machine learning methods. Specifically, the study aims to:

1. Design the architecture and workflow of an automated prosody analysis system.
2. Integrate speech preprocessing, feature extraction, and machine learning components into a functional prototype.
3. Demonstrate the feasibility of automating key stages of prosodic analysis using a manually annotated reference corpus.
4. Identify the opportunities and limitations of the proposed prototype to guide future system development and validation.

To achieve these objectives, the study addresses the following research questions:

RQ1. How can an integrated prototype be designed to automate the main stages of prosody analysis?

RQ2. What workflow and system architecture are suitable for combining speech preprocessing, feature extraction, and machine learning into a single analytical framework?

RQ3. To what extent does the developed prototype demonstrate the feasibility of automated prosody analysis using a manually annotated reference corpus?

RQ4. What limitations of the current prototype should be addressed in future work to improve its robustness and enable comprehensive quantitative evaluation?

RP has been selected as a reference point for teaching. When students aim to build a career in language – whether as a linguist, educator, or interpreter – their command of precise articulation and accurate perception is fundamental to professional competence. Moreover, in these professions, intonation serves as an indispensable tool that guides meaning, complements words and grammar, and organized spoken discourse. Despite the fact that some linguists question the status of RP as a model in L2 learning and there is a general shift towards acquiescence of other speakers’ accents of English ( Baratta, & Halenko, 2022), this course developers find it important to focus on RP. Mastering this type of articulation, that is recognised as a language norm, might enhance the learners’ intelligibility and help them fulfil their professional duties by serving as models of standard pronunciation. As professional linguists they need more than just producing intelligible utterances and recognizing regional accents as required by CEFR. They should be able to recognize, analyse, and explain the causes of such phonological phenomena as sound assimilation, reduction, palatalization, individual pronunciation irregularities, cross-linguistic interference with the L1, etc. Such focus on RP does not mean, however, that course instructors intentionally restrict exposure to other language varieties.

The course is structured across two semesters. It combines face-to-face sessions with guided independent study, providing students with flexibility while maintaining consistent academic support. In **the first term**, students focus on mastering sound articulation, transcription, and basic intonation models. The instructor-led sessions are complemented by self-paced practice, which includes both independent training and the use of the AI Pronunciation Trainer ( Lobato, 2025). This free service supports learners by offering immediate, phoneme-level feedback and visualizations via IPA transcription, encouraging repeated practice of individual sounds and sentence-level prosody. Although the functionality of the tool is limited, it is still helpful at the early stages, particularly for learners whose native language interferes with accurate sound production. The main drawbacks are the preset nature of the sentence bank and the rigid scoring system that rewards hyper-articulation without recognizing the natural use of reduction and assimilation. Thus, the usage of this AI-driven tool is complemented by corrective guidance from the professors during classes.

Another supplementary tool used at this stage of the course is Speechace (n.d.), a speech recognition platform designed for evaluating and giving feedback on pronunciation and fluency. Its AI-driven analysis allows learners to independently assess their pronunciation and share their results with instructors for progress tracking. The system provides a grade and a descriptive evaluation of the spoken input; however, it does not offer detailed analysis of suprasegmental features essential to our syllabus, such as rhythm, various types of stress, or intonation contours.

Speech analyzer Elsa Speak (2025) allows to evaluate both segmental and suprasegmental features of real-time user recordings. It provides scoring for vowel and consonant sounds and marks mispronounced phonemes in a color-coded transcript, which helps students visually track deviations from RP. The service also evaluates fluency through measurable metrics such as speaking pace, pause duration, and overall flow, and provides a graphical pitch line that reflects intonation control. Intonation feedback is expressed through both numeric ratings and visual indicators, supporting the development of pitch range awareness and rhythmical delivery. This detailed and accessible feedback makes this tool particularly useful for pronunciation assessment.

During **the second term**, instruction shifts toward more complex aspects of connected speech, including style-specific intonation and pitch variation. Students work extensively with authentic texts, which they first read aloud, then practise using the shadowing technique to refine pronunciation and prosody. As part of their work, students also transcribe the texts, mark phonetic phenomena such as assimilation, reduction, and intonation contours, and draw tonograms. This intensive work culminates in memorized recitation of the original texts. Through this progression students gradually develop the skills needed to create and perform their own phonetically accurate and communicatively meaningful texts.

None of the currently available AI-powered pronunciation assessment tools are suitable for the advanced prosodic analysis expected at the specialized university-level phonetics education. The available services focus primarily on segmental features and offer limited feedback on suprasegmental elements like stress placement and pitch movement. The students of the target group are trained to become professional linguists who will be required to produce utterances that reflect the phonetical features of the language norm, perceive and analyze intonation contours and rhythm in natural speech. The spectrograms, pitch traces, and timelines generated by Praat and other available tools enable students to visualize speech melody but do not provide sufficient data for detailed linguistic theoretically informed interpretation and instruction. Therefore, the main research question this project seeks to answer is how to develop an advanced automated prosody detection system based on machine learning.

## Materials and methods

The project implementation was organised in 3 steps.

The aim of the **preprocessing stage** was to generate a high-quality dataset suitable for training a machine-learning model that can provide automatically generated prosodical analysis. The fact that our learners are Russian native speakers prompted us to refer to extensive experience of Russian linguists who have established a unique scholarly tradition for analysing the prosodic aspects of a language. Intonational analysis in contemporary Russian phonetics studies relies extensively on formally defined symbol sets proposed by phoneticians ( Antipova, et al., 1985; Mitrofanova, 2012; Vereninova, 2011). Some examples of intonation markers and graphical representations of tonograms used for teaching pronunciation are illustrated in Figure 2. In this study, the researchers draw upon this scheme with slight modifications that allow us to accommodate both the instructional needs of the above-mentioned practical phonetics course and the algorithmic requirements of the system.

**Figure 2.**
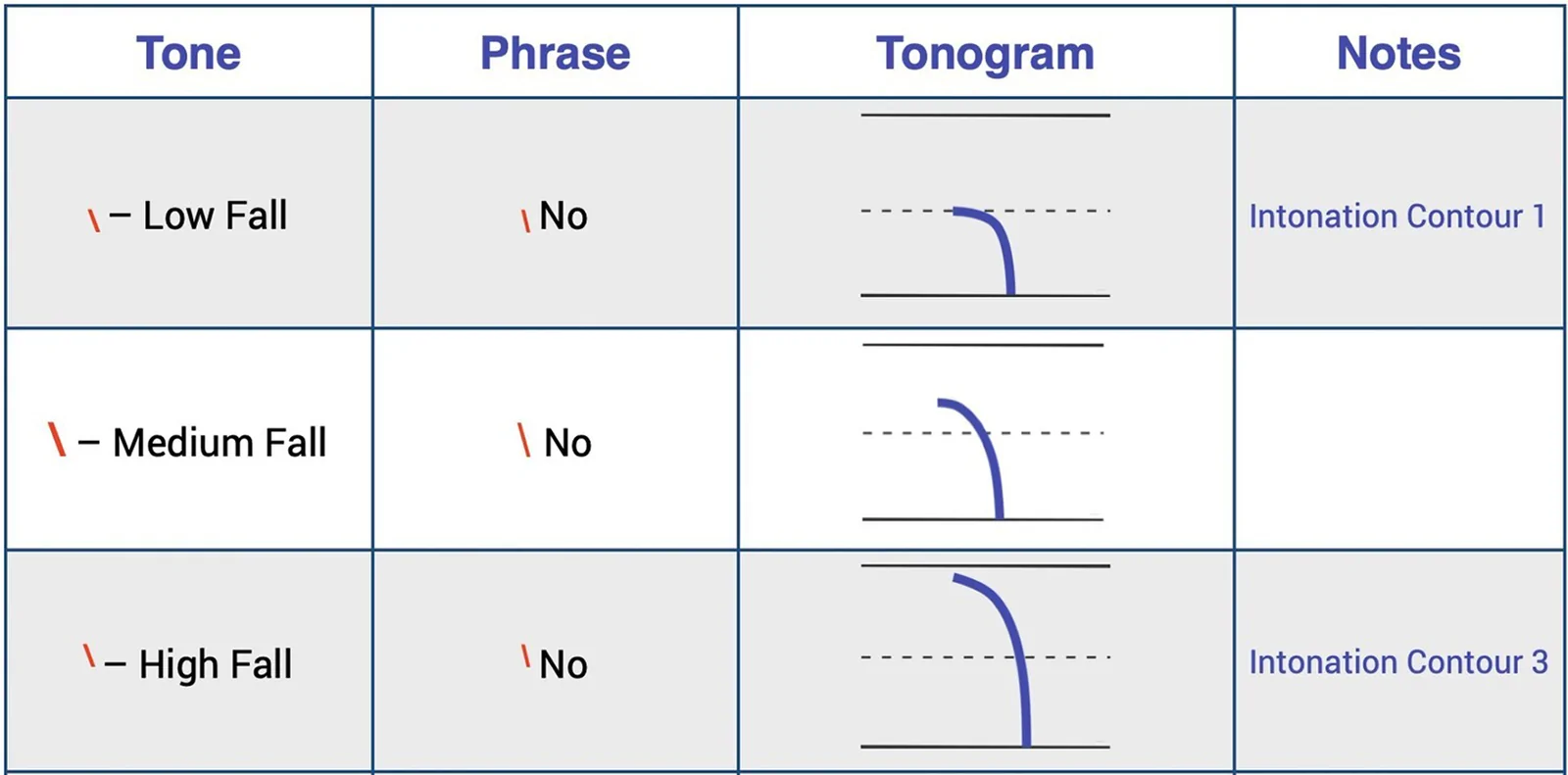
Examples of intonation markers and pitch contour diagrams.

This set of symbols was used to examine forty-seven audio samples. Each recording was analyzed, fully transcribed, and intonational markers were placed in alignment with the speakers’ prosodic patterns. Figure 3 illustrates a part of such text with the completed intonational analysis.

**Figure 3.**
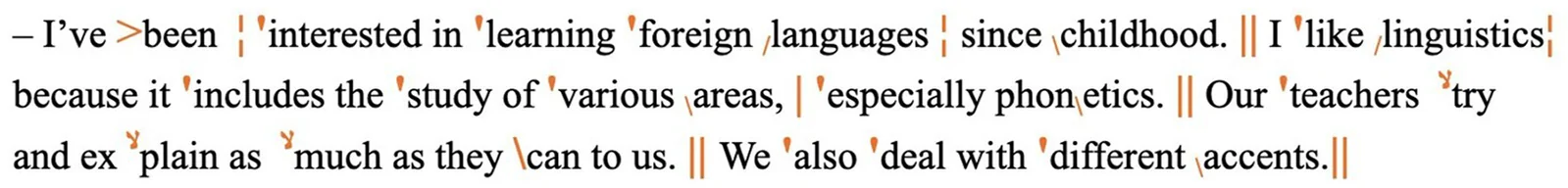
An example of text with phonetic markers reflecting the speaker’s prosody.

It is worth noting that the manual annotations were independently prepared by three linguists holding PhDs in Applied Linguistics using this system of intonational analysis. Following the independent annotation stage, the experts reviewed and reconciled any discrepancies through discussion to establish the final reference annotations used for prototype development. As the primary objective of this phase of the project was the development of a proof-of-concept prototype, formal inter-annotator agreement statistics were not calculated and are planned for future validation studies.

The **data processing stage** involved applying software libraries suited for analysing the acoustic properties of audio files. Because the audio recordings constitute audio of unstructured modality, the standard data-science stack ( *pandas, NumPy, Matplotlib*) was augmented with *librosa.* Data exploration and preparation were performed in *JupyterLab 4.3.6* running *Python 3.13.* Several package versions were employed, including *librosa 0.11.0* for handling audio input, time-series, slicing, mel-spectrogram construction, and on-screen display via *librosa.display*; *Matplotlib 3.10.1* for exploratory plots; *NumPy 2.1.3* for numerical arrays; and *pandas 2.2.3* for tables. Background noise was suppressed with *noisereduce 3.0.3.*

The **model selection stage** involved training machine learning models and evaluating their accuracy. For modelling, *scikit-learn 1.6.1* was utilised to train classical algorithms, while *PyTorch 2.3* (CPU-only) was employed for the convolutional network. Each training iteration was tracked with a *tqdm* progress bar. Initially, annotation dashboards were created with *Bokeh*, *HoloViews*, and *Panel.* Eventually, they were replaced with *LabelStudio 1.17.0* running in *Docker* for manual verification. The prototype comprised several containerised services, including *FastAPI*, *RabbitMQ*, *MinIO*, *PostgreSQL*, and a *React SPA*, which communicated through *Docker Compose* and were prepared for deployment on *Kubernetes.* The system architecture was documented in *PlantUML* and visualised using *C4 Model* diagrams.

The project architecture presented in Figure 4 employs a modular, micro-service-styled design in which data ingestion, storage, task routing, and presentation layers are separated into distinct components, following the principles described by Newman (2021). To document this design clearly, the C4 Model ( Brown, 2023) was used as the guiding framework for architectural diagrams, as it provides hierarchical views (context, containers, components, code) that align with our service-based architecture. The main advantage of C4Model lies in its support for incremental elaboration, as not all layers need to be formalised simultaneously.

**Figure 4.**
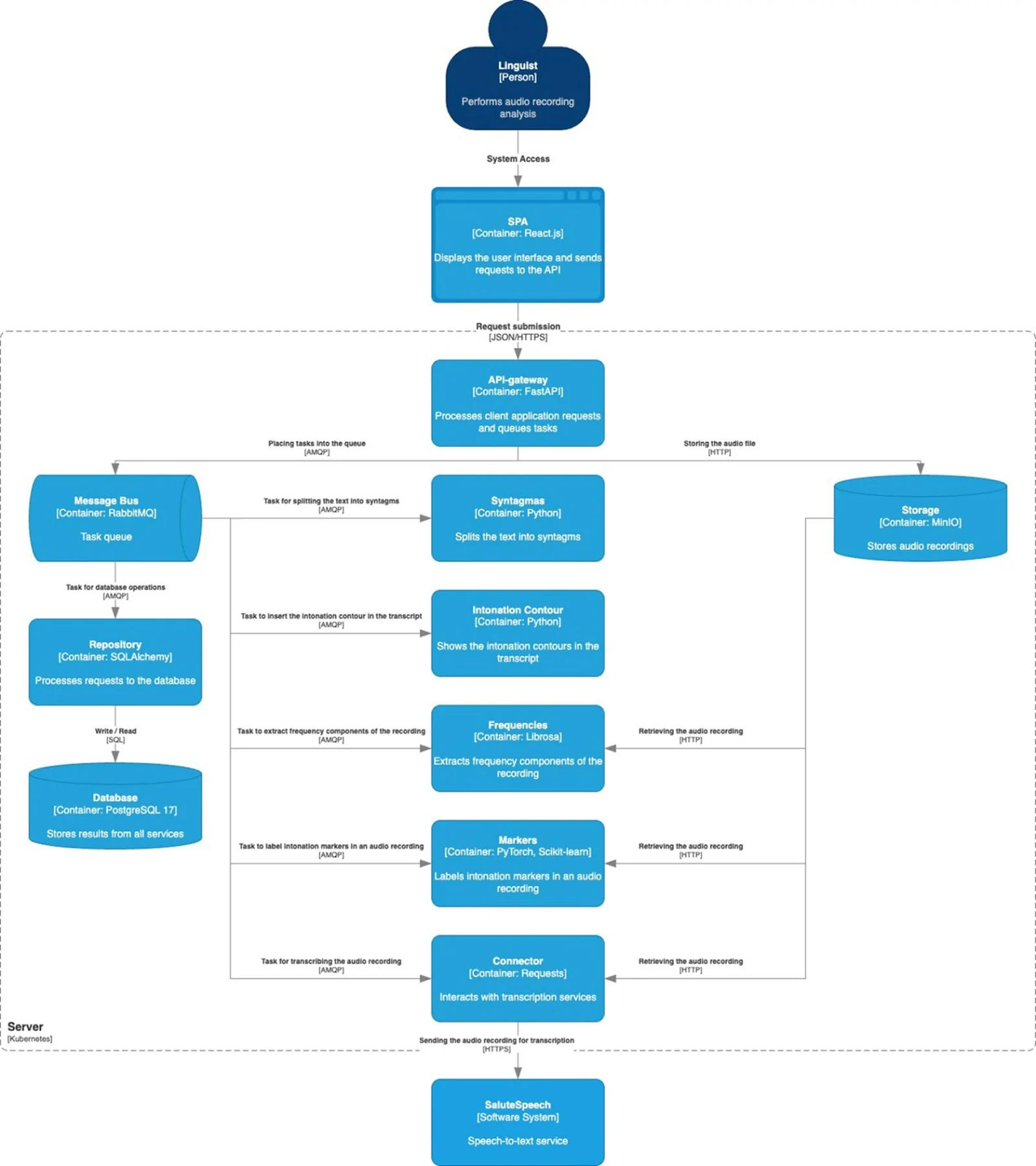
Container-level view of the software architecture for intonation processing.

At the container level, the C4Model depicts a micro-service back-end in which each functional task is isolated in its own Docker container. Inter-service communication is managed by a *RabbitMQ* message queue, with all connecting arrows annotated as AMQP. When a service needs to read or write an audio file or its derived spectrogram, it issues a simple HTTP request to *MinIO*, an S3-compatible object store. HTTP label is deliberately retained for clarity because S3 may refer either to the storage engine or to its protocol.

Database operations are routed through a specialized repository service that submits SQL queries to a PostgreSQL server. The connector is annotated SQL because the language of the request conveys more meaningful information than the transport protocol, and that mirrors the way of tagging AMQP for message exchange. The remaining containers that represent microservices responsible for the logic of the application are described in Table 1.

**Table 1.** Functional roles of containers in the back-end architecture.

Container	Its main function
Connector	Sends audio to SaluteSpeech (speech-to-text) and posts the transcript back to the queue.
Frequencies	Computes and plots the recording’s frequency spectrum.
Markers	Applies the ML model to label intonation markers.
Syntagmas	Splits the transcribed text into syntagmatic units.
Intonation Contour	Aligns predicted markers with the transcript, producing a pitch-contour overlay.

The steps below trace the path of an audio recording through the containers shown in Figure 4.

1. The user uploads an audio file through the web interface ( *SPA*).
2. The file object reaches the *API-Gateway* endpoint.
3. The *API-Gateway* stores the file in the object repository ( *Storage*).
4. Using the *Message Bus*, the gateway enqueues three work items, routing them to *Connector*, *Frequencies*, and *Markers* accordingly.
5. *Connector* retrieves the transcription task, downloads the audio file from *Storage*, and sends it to *SaluteSpeech.* When the transcript is returned, *Connector* posts a new task for *Syntagmas.*
6. *Frequencies* container processes its queue entry, downloads the audio file, generates spectral plots, saves the plots back to *Storage*, and queues a *Repository* task that carries the plot IDs.
7. *Markers* container downloads the audio file, runs the ML model to identify intonation markers, and publishes the results to *Repository* via *Message Bus.*
8. *Syntagmas* container retrieves the segmentation task and transcript, splits the text into syntagmatic units, and enqueues the segmented output for Intonation Contour.
9. *Intonation Contour* combines the marker data (retrieved via *Repository*) with the segmented text, creates the final prosodic overlay, and sends the package to *Repository.*
10. *Repository* executes all read/write requests against *PostgreSQL.*
11. Once every task for the given recording is complete, *API-Gateway* notifies the SPA over *WebSocket*; the client then requests the results.

## Results

### Data-processing stage

At the preprocessing stage, as was mentioned earlier, forty-seven audio samples were transcribed and annotated to serve as input for machine learning tasks.

The initial data-processing stage involved applying software libraries suited to analyse the acoustic properties of audio files. As the recordings constituted unstructured data, the standard data-science stack ( *pandas*, *NumPy*, *Matplotlib*) was augmented with *librosa.* Data exploration and preparation were conducted in *JupyterLab 4.3.6* running *Python 3.13.* The employed package versions were: *librosa 0.11.0*; *Matplotlib 3.10.1*; *NumPy 2.1.3*; and *pandas 2.2.3.* With *librosa.load* function, two essential parameters were extracted from each audio track: an array representing the waveform and the sampling frequency which is set by default to 22.050 Hz. This function accommodates full-length loading as well as partial loading through the optional duration and offset parameters. Figure 5 illustrates the loading command and the data structures it returned.

**Figure 5.**
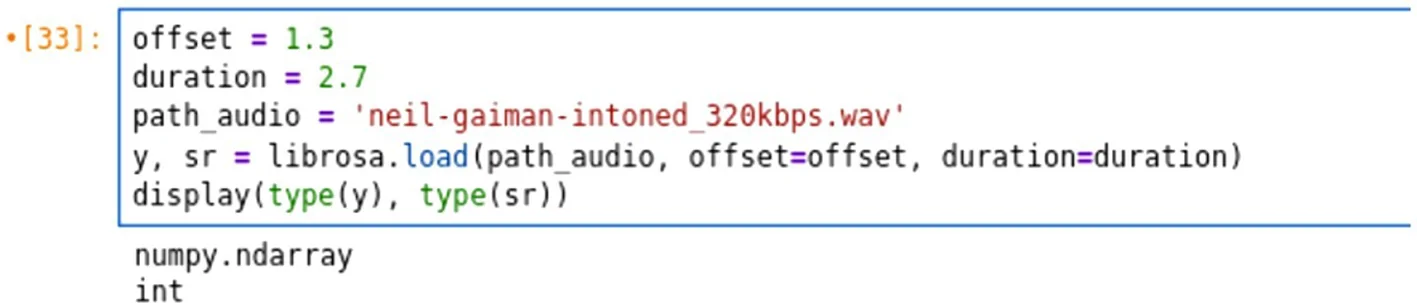
*Librosa* workflow in *JupyterLab* illustrating the output of the load method.

Data returned by *librosa.load* were used to create visualisations that revealed the characteristics of the analysed waveform. Similar to how the *head()* method in a *pandasDataFrame* offered a preliminary view of a dataset, *librosa.display.waveshow* provided a quick visual inspection of the audio signal a sample of which is shown in Figure 6.

**Figure 6.**
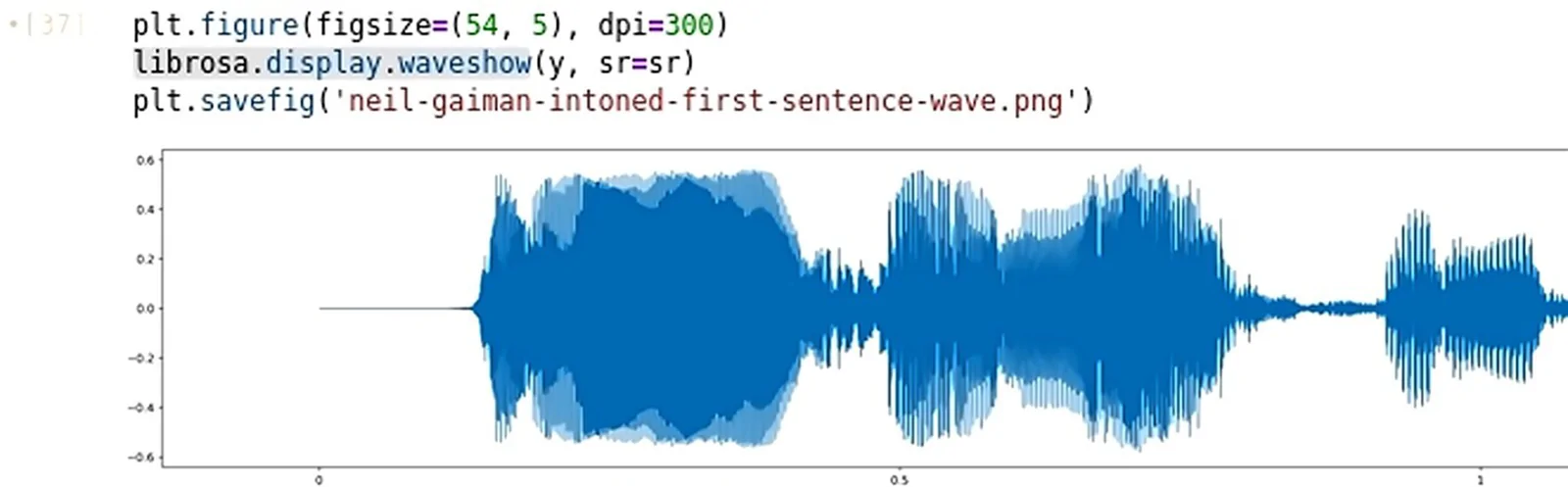
Displaying and exporting an audio waveform using visualization tools.

For the current investigation, a logarithmically scaled spectrogram, rendered as a heat map, provided the most informative visual support. As can be seen in Figure 7, in this representation, the hottest regions – corresponding to the highest decibel values – form patterns that resemble the tonograms phonetician for intonation annotation.

**Figure 7.**
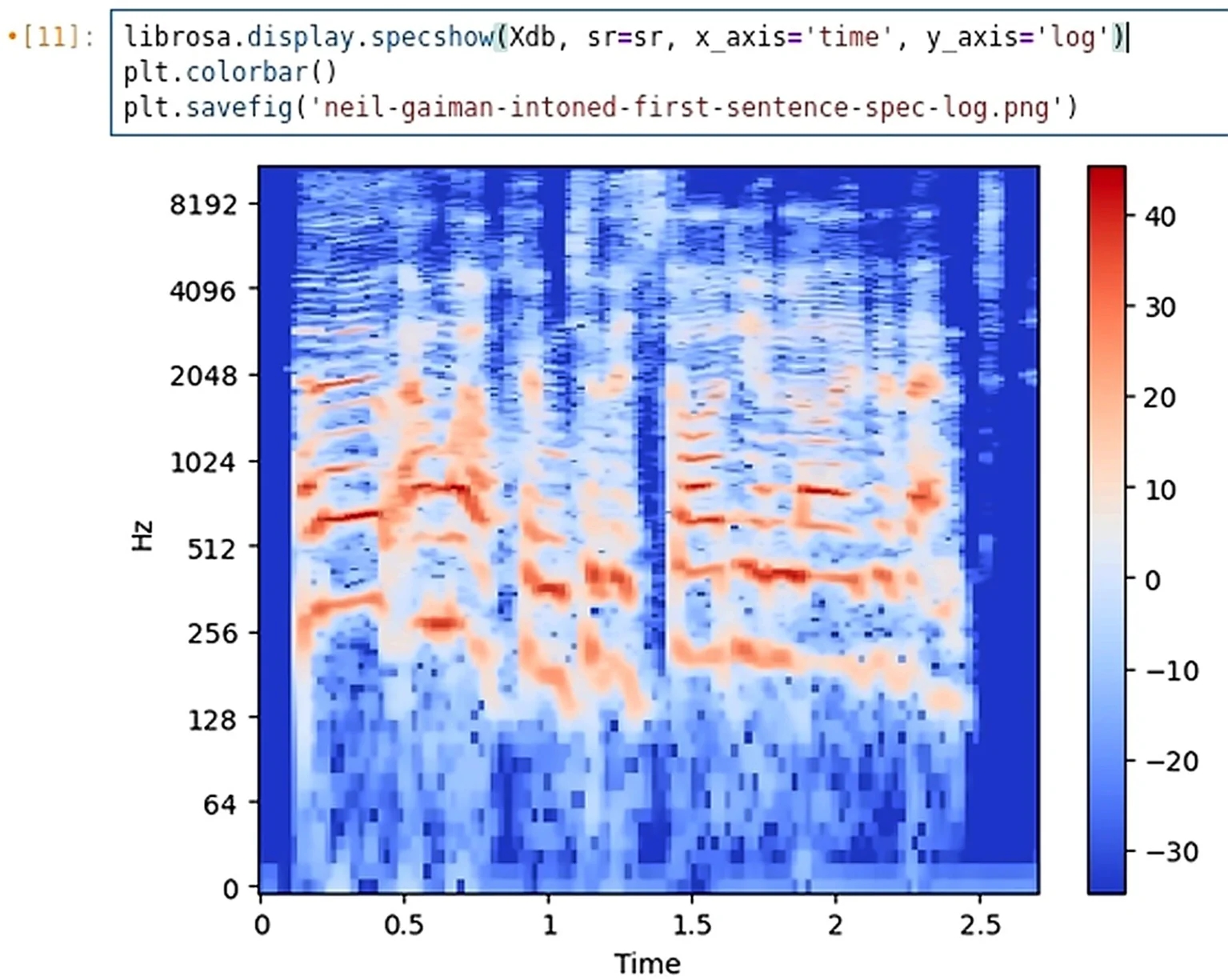
Spectrogram of the selected audio excerpt plotted on a logarithmic scale.

After the trial with separate recordings, a Python script was developed to automatically extract an acoustical dataset from all forty-seven audio files. Each record included the following attributes: timestamp, amplitude, harmonic signal component, percussive signal component, denoised amplitude, sample number, recording ID, aggregate duration of the recording, sampling frequency.

Special attention was paid to the noise-filtered amplitude and the sample index. Background noise was suppressed with *noisereduce* 3.0.3 (function *reduce_noise*). It allowed to process raw-amplitude arrays and the sampling frequency. Then, *librosa.effects.split* was applied to the denoised signal. This utility segmented a *NumPy* amplitude vector wherever the sound-pressure level dropped below the silence threshold (60 dB by default). A sample code for denoising an audio signal is presented in Figure 8. The combined use of *noisereduce* and *librosa.effects.split* produced a noise-free waveform and temporal segments, which were later reused for raw, harmonic, or percussive amplitude streams. Noise removal resulted in silence gaps at previously noisy segments.

**Figure 8.**
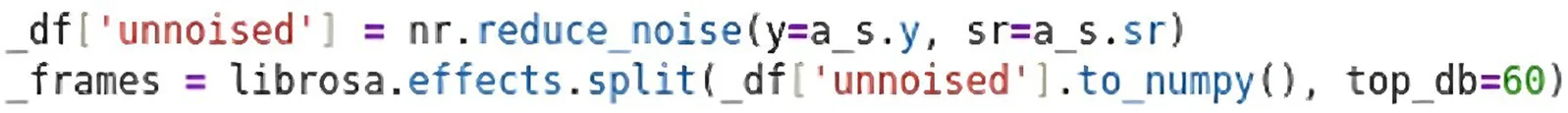
Code snippet demonstrating the use of *noisereduce* and *librosa.effects.split* (threshold = 60 dB).

As a result, the script generated logarithmically scaled spectrograms for each of the forty-seven recordings and exported them as 300 dpi *PNG* images that were compressed into a 413 MB ZIP folder. The dataset was stored in *Parquet* format, resulting in a reduction in size from 6.8 GB (CSV) to 1.9 GB. This format was selected due to its high efficiency and broad compatibility. Specifically, *Parquet* can be seamlessly integrated with both *pandas* and *PySpark*, and it can be efficiently utilized as a data source within the *ClickHouse* environment. Its column-oriented architecture not only optimizes storage space but also facilitates column-wise data retrieval and predicate push-down filtering during data loading, thereby improving overall performance and analytical efficiency.

After audio preprocessing, the dataset was segmented into a primary subset containing filenames, sampling frequencies, and file durations, and secondary subsets comprising raw, denoised, percussive, and harmonic amplitude data.

The subsequent step of data processing involved normalization and intonation labelling. Normalization was performed using the *StandardScaler.fit_transform* method from *scikit-learn 1.6.1.* For labelling, a *CategoricalDtype* was defined in *pandas*, with categories corresponding to the specified intonation contours ( Antipova et al., 1985). This categorical type was then assigned to a newly added field, *mark*, where the default value *plain* indicated the absence of a distinctive pitch contour, as illustrated in Figure 9.

**Figure 9.**
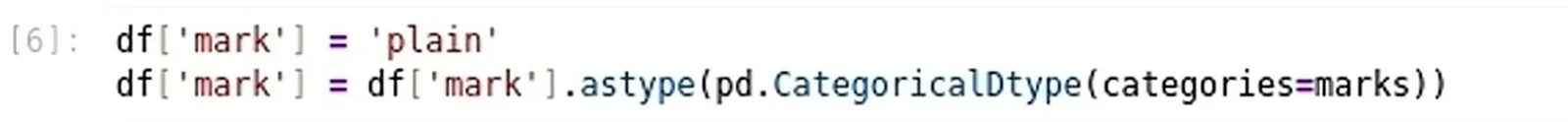
Code snippet for defining intonation categories.

After defining the intonation categories and adding the *mark* field, the labelling process was carried out according to a nine-step pipeline.

1. Retrieving the normalized data corresponding to the selected sample and file from the relevant dataset.
2. Generating a spectrogram with an accompanying playback cursor.
3. Loading the primary dataset into memory.
4. Identifying the segment of interest within the primary dataset.
5. Verifying the audio by cross-checking the spectrogram with the marked text.
6. Determining the temporal boundaries of the intonation marker.
7. Assigning intonation labels to all data points within the defined interval.
8. Reviewing the newly assigned labels to ensure consistency.
9. Saving the updated primary dataset.

The implementation of the approach described above required the use of additional libraries, namely *bokeh 3.7.2; holoviews 1.20.2; panel 1.6.2; scipy 1.15.2.* As a result, labelling one file took 6–8 hours on average. The process was not only time-consuming but also revealed a critical memory issue because *pandas* stored data frames in *RAM*, and *Python* did not trigger the *garbage collector* automatically when a variable was reassigned. In a series of labelling cycles, the system exhausted its available RAM and swap space, which caused the operating system to terminate *JupyterLab.* Preventive measures required continuous monitoring of memory consumption and either explicit garbage collection calls or regular *JupyterLab kernel* restarts.

Due to these multiple factors that slowed the process, it was suggested to replace the toolset with *Label Studio 1.17.0* to complete the data-processing stage. The service’s web interface was modified, including adjustments to waveform height, playback controls, and default zoom. Annotation was conducted in *Chromium 136.0.7103.113.* All audio files and intonation labels were uploaded in a project in *Label Studio.* The main features of this annotation process are shown in Figure 10. The new environment reproduced the stages formerly executed in *JupyterLab,* reducing the average labelling time to 1 hour per file as resource monitoring was not required.

**Figure 10.**
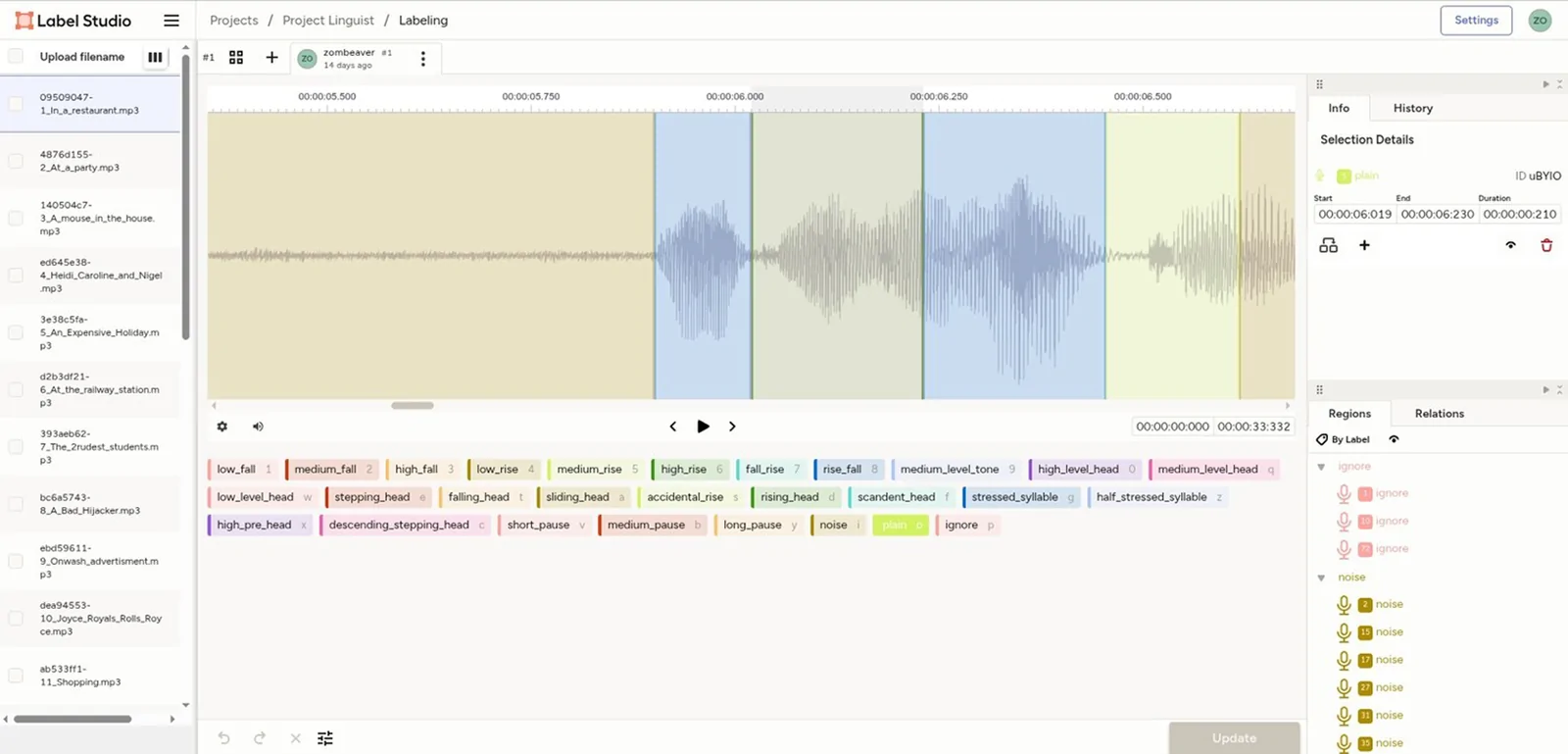
*Label Studio* annotation process.

### Model selection stage

Having processed the raw audio into an annotated corpus of 882 manually labelled audio clips with durations from 0.5 seconds to 4 seconds, researchers could shift from data procession to model selection. The objective of this stage was to determine which machine learning approach could replicate manual labelling.

To facilitate comparison, the algorithms were grouped into two categories – classical models and deep-learning models. Classical models refer to methods that rely on algorithms other than neural networks, such as *k-nearest-neigbours*, *support-vector machines*, and single-layer perceptron variants. Deep-learning models rely on multi-layer neural architectures. Such grouping allowed us to determine whether observed accuracy gains were a consequence of the learning architecture or the set of descriptors supplied to the models.

Model generation and testing were automated to perform a hyper-parameter grid search and log performance metrics for each configuration. Each run recorded the model ID, the hyper-parameter configuration, and the accuracy score. Experiments were conducted in *JupyterLab* with a *tqdm* progress bar on an *AMD Ryzen 7 5700G* processor with 8 cores, 16 threads, and a 4.6 GHz turbo frequency.

All classical models were trained in *scikit-learn 1.6.1* using four low-level acoustic descriptors: mel-spectogram amplitudes, mel-frequency cepstral coefficients (MFCCs), spectral slope, and zero-crossing rate. The *GaussianNB* was selected as the baseline algorithm because of its minimal hyper-parameter set. It produced the lowest pilot accuracy, reaching 0.13208. In total, 4909 classical configurations were evaluated: *MLPClassifier* (4332), *KneighborsClassifier* (380), *RandomForestClassifier* (150), *LogisticRegression* (36), Support-Vector Classifier SVC (10), and *GaussianNB* (1). An overview is provided in Table 2.

**Table 2.** Hyperparameters and their tested ranges for classical machine learning classifiers in *scikit-learn.*

Model (skirit-learn class)	Hyper-parameter	Values/Range
GaussianNB	–	–
KNeighborsClassifier	*n_neighbors*	5–100
	*weights*	uniform, distance
	*p* (Minkowski metric)	1,2
Support-Vector Classifier (SVC)	* decision_function_shape *	ovo, ovr
	*kernel*	linear, poly, rbf, sigmoid, precomputed
LogisticRegression	penalty	l1, l2, elasticnet lbfgs, liblinear, newton-cg, newton-cholesky, sag, saga
	solver	l1, l2, elasticnet lbfgs, liblinear, newton-cg, newton-cholesky, sag, saga
	multi_class	multinomial, ovr
RandomForestClassifier	n_estimators	100–1000 (step 100)
	max_depth	10–50 (step 10)
	criterion	gini, entropy, log_loss
Multilayer Perception (MLPClassifier)	solver	adam, lbfgs, sgd
	max_iter	2000
	alpha	0.00001, 0.0001, 0.001, 0.01
	hidden_layer_sizes	2 layers; 10–100 (step 5)

*GaussianNB* yielded the lowest performance, with an accuracy of approximately 0.13. *KNeighborsClassifier* achieved a peak accuracy of 0.31638 across multiple configurations, with the number of *n_neighbors* being the primary driver of computational cost and performance variation. *SVC* produced the same accuracy (0.31638) under *poly* and *rbf kernels.* *LogisticRegressio*n achieved an accuracy of 0.32203 when trained with *saga solver* and *multinomial* option. The strongest results were obtained through *RandomForestClassifier.* An accuracy of 0.37288 was achieved with *n_estimators* = 600 and also with *n_estimators* = 900 (using max_depth = 10, criterion = ‘gini’). Since the larger forest incurs higher computational costs without improving performance, the 600-tree configuration is preferred. The *MLPClassifier* reached an accuracy of 0.36158 with *solver* = *lbfgs*, *alpha* = 0.01, and a 2-layer topology of (50, 10) neurons. Experiments at the largest hidden-layer sizes saturated all CPU threads on the hardware which limited stability. In general, no classical model exceeded 0,38 accuracy. Feature-only approaches proved insufficient for accurately capturing prosodic markings. Consequently, the experiment was shifted to convolutional neural networks (CNNs), which can automatically learn relevant features from raw data.

The CNN developed for this experiment was implemented in PyTorch as a subclass of *nn. Module* ( Figure 11). CNN is implemented in PyTorch as a subclass of *nn. Module* (see Figure 11). The network comprises up to five identical blocks, each applying a 5x5 filter to the mel-spectrogram, normalizing the output, and then passing the result through a non-linear activation function: either ReLU or LogSigmoid. After the last block, the network compresses the entire time-frequency representation into a vector and feeds this vector into a linear layer that produces 19 output scores - one for each intonation class. In this way, the CNN learns to transform a colored mel-spectrogram into a set of probabilities for the intonation labels.

**Figure 11.**
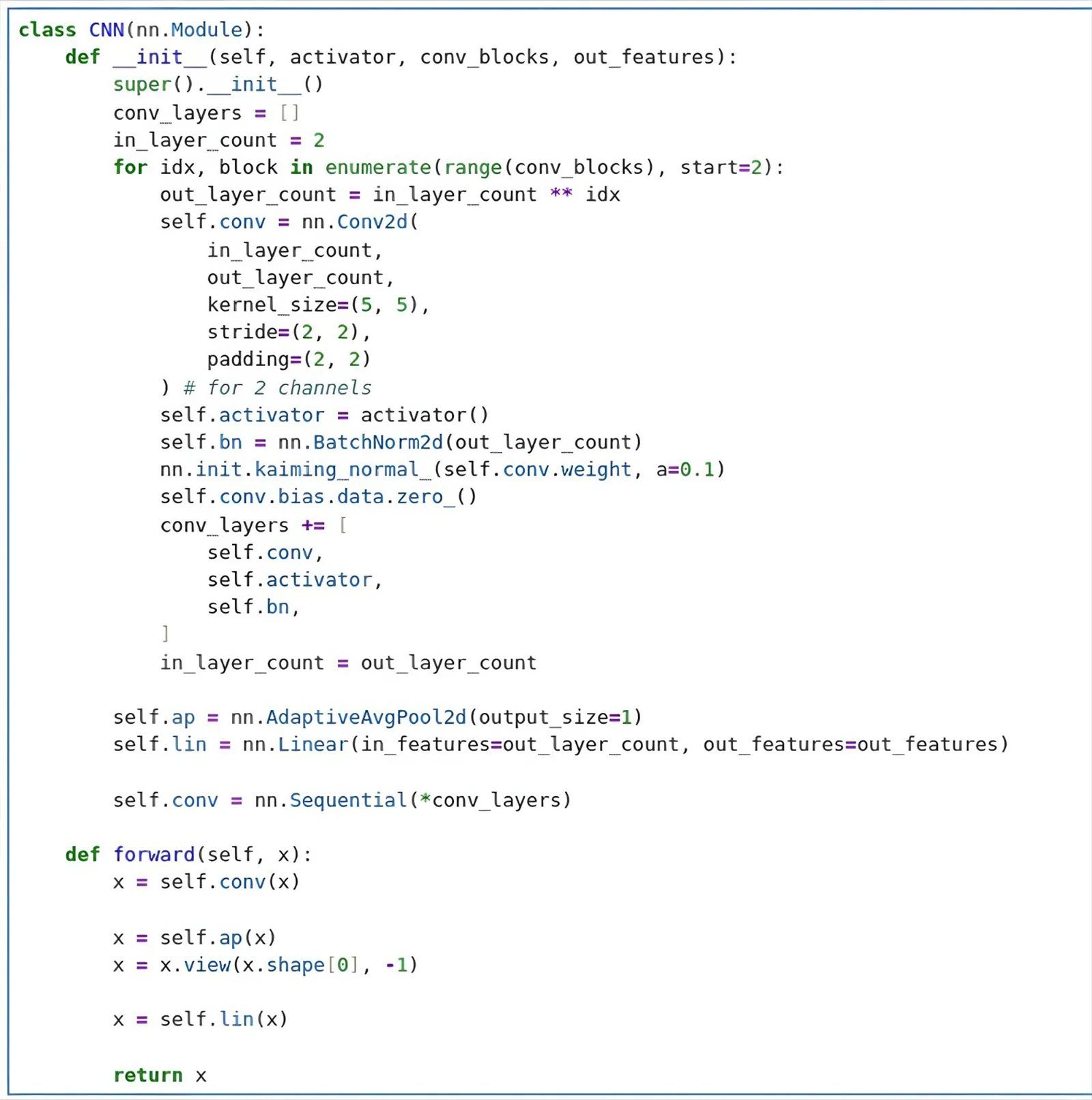
Code snippet defining a CNN.

To evaluate the model’s performance under different configurations, six key training parameters were systematically varied, as summarised in Table 3.

**Table 3.** Hyperparameter grid defining the range of values explored during CNN training.

Hyper-parameter	Values tested
Activation function	ReLU, LogSigmoid
Loss function	MSELoss, CrossEntropyLoss
Optimiser (update rule)	SGD, Adam
Learning rate	0.1, 0.01, 0.001
Number of convolutional blocks	1–5
Training epochs	5–100 (in steps of 5)

Systematically varying these hyper-parameters produced 120 distinct CNN configurations. Each configuration was trained and evaluated under identical conditions to isolate the factors contributing to accuracy improvements such as network depth, choice of optimiser, and duration of training. As illustrated in Table 4, the highest accuracy of 0.45455 was achieved by two configurations, both employing the Adam optimiser but differing in loss function (MSELoss versus CrossEntropyLoss), learning rates, and number of convolutional blocks. The accuracy margin between these top-performing models and the next best configurations exceeded three percentage points, indicating that model depth and learning rate exert a decisive influence on performance.

**Table 4.** Hyper-parameters of the two best-scoring CNNs.

Huper-parameter	ReLU/Adam	LogSigmoid/Adam
Learning rate	0.001	0.100
Number of convolutional blocks	2	1
Epochs	25	70
Accuracy	0.45455	0.45455

It can be concluded that the CNN applied to the audio data represented a deep-learning approach within the broader domain of natural language processing. Although it outperformed the classical, feature-based classifiers, even the best-performing CNN configurations did not achieve sufficient accuracy for reliable automatic intonation marking.

## Discussion and conclusions

Mastering pronunciation remains an important aspect of foreign language learning because it helps speakers convey shades of meanings and serves as a strong non-verbal clue for comprehension. Modern AI-powered tools are often capable of serving as a pronunciation model and providing considerable assistance in analysing prosodical aspects of utterances produced by learners. However, when it comes to a more profound analysis required by professional linguistics or students who are training to be phoneticians, such functionality does not suffice.

The described project is aimed at creating a platform that could use the principles of machine learning to automatically generate prosodic analysis that extends beyond individual sound segments and reflects suprasegmental aspects of prosody including rhythm, intonation, and phrasal stress. The experiment involved training and evaluating two types of models: classical machine-learning classifiers and a deep convolutional neural network (CNN). All models were trained on 882 annotated audio fragments containing prosodic information. The classical models relied on manually engineered prosodic features, requiring detailed contour statistics derived from the speech signal to represent intonation patterns. In contrast, the CNN operated directly on mel-spectrograms, learning relevant features automatically from the audio data. To improve generalization, the CNN was trained on an augmented dataset that included pitch shifts, noise overlays, and time-frequency masks. Both model types were trained and validated under identical conditions to compare their effectiveness in automatic intonation marking.

The experimental results showed that the CNN model achieved an accuracy of 0.45455, while the best classical alternative, *RandomForestClassifier*, reached 0.37288. This represents an approximate 22% relative improvement in classification accuracy, confirming the advantage of a deeper architecture. However, despite this gain, the performance remains insufficient for reliable automatic intonation marking.

Overall, the transition from classical, feature-based classifiers to convolutional neural networks (CNNs) led to a measurable improvement in accuracy and demonstrated the potential of deep learning for prosodic analysis. However, despite outperforming traditional models, the CNN configurations tested in this study did not yet achieve sufficient reliability for practical intonation marking. These results highlight both the promise of deep-learning approaches and the need for further optimisation and data enrichment.

In light of the identified prototype limitations, a roadmap for further improvement has been developed. The first stage of improvement will involve finalising an authenticated minimum viable product (MVP) capable of ingesting audio, storing it in MinIO, routing processing tasks through RabbitMQ, and displaying basic intonation labels in the single-page application (SPA). The subsequent development stages will introduce stress detection, tone-scale plotting, and script-based annotation, followed by the integration of rhythm analytics to enhance prosodic analysis capabilities. A further step will involve releasing a containerised version of the tool with user control features, PDF export, change-approval tracking, and learning management system (LMS) integration to support deployment and classroom use.

An accompanying development task is to expand the training corpus. This involves the continuous addition of new audio-text pairs manually labelled with the same 19 intonation markers used in the experiment. For each text already included in the experimental dataset, multiple readings will be collected from new speakers. This procedure will introduce diversity in timbre and speech rate while preserving the prosodic pitch contour. The enlarged corpus is expected to provide the statistical power necessary to improve accuracy beyond the current 0.46 level.

As this study presents the development of a prototype for automated prosody analysis, its primary focus was on the system’s design and implementation. Although a manually annotated reference corpus was created during the preprocessing stage of machine learning, a comprehensive quantitative evaluation of the prototype’s performance was beyond the scope of the present work. Although the reference corpus was independently annotated by three expert linguists and reconciled through discussion, formal inter-annotator agreement statistics were not calculated. Future studies will validate the system against the annotated corpus using established performance metrics, including accuracy, precision, recall, and F1-score, and will incorporate additional statistical analyses to provide a rigorous assessment of the prototype’s effectiveness.

Despite these planned improvements, several limitations of the current project should be acknowledged. Although the suggested tool incorporates additional functionality, it might be of interest to a limited group of linguists and speech analysts. English language teachers might find it less appealing since tonogram is not widely applied in contemporary pedagogical practice; however, its potential use in linguistic research might lead to valuable observations. Besides, it might find its place in the language teaching classroom designed for philologists, interpreters, and language teachers.

Moreover, the development of an automatic prosody detection tool aligns with the principles of the Sustainable Development Goals by enhancing access to quality education and promoting innovation and infrastructure. Such technology provides learners with equitable opportunities to receive consistent and personalized feedback on their speech, reducing barriers caused by limited instructional time or resources. By supporting effective language learning and communication skills, automatic prosody detection tools contribute to lifelong learning, employability, and social inclusion, thereby reinforcing broader commitments to sustainable and inclusive development in a digitally driven society.

## Ethical statement

This study was approved by the NUST MISIS Ethics Committee, and written informed consent was obtained from all participants prior to data collection. No reference number was assigned.

## Data Availability

No underlying data are associated with this article.

## Software availability

Source code available from: https://github.com/DoctorKutuzov/ProjectLinguist; https://github.com/DoctorKutuzov/project-linguist-jupyter; https://github.com/DoctorKutuzov/project-linguist-api-gateway

Archived software available from: https://doi.org/10.5281/zenodo.19186923; https://doi.org/10.5281/zenodo.19186941; https://doi.org/10.5281/zenodo.19186929

License: MIT License.

The GitHub repository associated with this study provides access to the codebase developed to date. However, the software as a complete standalone tool is not yet finalized, since development is still in progress. As noted in the article, the present study describes the current stage of development rather than a fully completed software product.
