# Supplementary material for: Towards Reliable Prosody Detection in Teaching Practical Phonetics: A Sustainable Digital Approach
Source: F1000Res. 2026 Oct 10;15:648. Originally published 2026 Apr 30. [Version 3] doi: 10.12688/f1000research.178913.3 (PMC13673075; doi:10.12688/f1000research.178913.3)
Supplement: Peer review history

# Reviewer report: Reviewer response for version 3

**DOI:** 10.5256/f1000research.211718.r528633

**Published:** 2026-10-21

**Authors:** Dony Marzuki

**Affiliations:** 1 Politeknik Negeri Padang, Padang, Indonesia

**My Second Comment:**

 I appreciate the writers' response to the initial comments. The additional analyzes make the paper stronger, and the rewritten manuscript is clearer in a number of ways. I've included my final remarks below:

*First comment (ethics).*

 My problem is addressed in the updated ethics statement. One more thing though, since the study included voice recordings of children, it would be better if a brief description of how these recordings were stored and safeguarded (e.g., where they were kept, who had access, whether they were de-identified or anonymized, and how long they will be kept or when they will be deleted) is provided. *The second comment (theoretical framing).*

 I still found it challenging to find the corresponding modifications in the updated manuscript. Could the authors ensure that the document text is as explicit as the explanation provided in the answer letter and specify the precise sections and page or line numbers where the adjustments were made?

*The third comment (duration of intervention and null findings).*

**My most unresolved concern is this** **one**. The authors point out in their response that not every result was null. I acknowledge this, but my initial request was for a discussion of the potential causes of the reported null results. I would request that the writers broaden the discussion to take into account different interpretations, such as: Is a three-week intervention long enough to yield a discernible improvement in the desired outcomes? The answer clarifies that the course schedule determined the duration. Although this is a reasonable practical limitation, it is not a sufficient explanation on its own. Please cite previous intervention studies of a similar scope to support the chosen length; if not, explicitly identify it as a limitation and explain how it might have impacted the outcomes.

 Whether the ASR-trained group's improvement is due to increased familiarity with the ASR technology or a true improvement in the targeted skill. This group did not improve on the other measures, but they did improve on the ASR-scored measures. This pattern is consistent with a familiarity or practice effect, and I believe it has to be openly addressed, either by discussion as a substitute explanation or by further investigation if the data permit it.

*The fourth (Methods and analysis).*

 Confidence intervals, ICC, and Bland-Altman analyzes are added, which goes above and above what I requested and enhances the document. Two minor points: first, so that readers can follow the adjustment, please clearly identify the sample-size inconsistency in the initial contribution and how it was fixed. Second, if there is published data supporting the efficacy of the ASR system as an assessment tool, please cite it. This is especially important when it comes to child speakers or learners with comparable profiles. It should be highlighted as a limitation if it doesn't.

*The fifth (Reproducibility).*

 I appreciate the extra information. I would advise the authors to publish the scoring rubric and the intervention materials (or a representative sample) as an appendix or supplemental file, or to provide a link to their availability, in order to further enhance replication.

Is the work clearly and accurately presented and does it cite the current literature?

Partly

If applicable, is the statistical analysis and its interpretation appropriate?

Partly

Are all the source data underlying the results available to ensure full reproducibility?

No

Is the study design appropriate and is the work technically sound?

Partly

Are the conclusions drawn adequately supported by the results?

Yes

Are sufficient details of methods and analysis provided to allow replication by others?

Partly

Reviewer Expertise:

EFL/ESL instruction, speech fluency, strategy training, autonomous learning

I confirm that I have read this submission and believe that I have an appropriate level of expertise to confirm that it is of an acceptable scientific standard, however I have significant reservations, as outlined above.

# Reviewer report: Reviewer response for version 3

**DOI:** 10.5256/f1000research.211718.r528632

**Published:** 2026-10-15

**Authors:** Abdul Hameed Panhwar

**Affiliations:** 1 University of Sindh, Hyderabad, Pakistan

Accepted without changes.

Is the work clearly and accurately presented and does it cite the current literature?

Yes

If applicable, is the statistical analysis and its interpretation appropriate?

I cannot comment. A qualified statistician is required.

Are all the source data underlying the results available to ensure full reproducibility?

Yes

Is the study design appropriate and is the work technically sound?

Yes

Are the conclusions drawn adequately supported by the results?

Yes

Are sufficient details of methods and analysis provided to allow replication by others?

Yes

Reviewer Expertise:

NA

I confirm that I have read this submission and believe that I have an appropriate level of expertise to confirm that it is of an acceptable scientific standard.

# Reviewer report: Reviewer response for version 2

**DOI:** 10.5256/f1000research.205064.r517667

**Published:** 2026-09-07

**Authors:** Anfal Sabeeh Hamood

**Affiliations:** 1 University of Baghdad, Baghdad, Baghdad Governorate, Iraq

This revised manuscript presents the development of a proof-of-concept automated prosody analysis system intended primarily for advanced practical phonetics and linguistics education. The study combines manual prosodic annotation, audio preprocessing, machine-learning classification, convolutional neural networks, and a containerised software architecture. The dataset was derived from 47 recordings and subsequently segmented into 882 manually labelled audio clips. Several classical machine-learning models were compared with CNN-based approaches. The best Random Forest classifier achieved an accuracy of approximately 0.373, whereas the best CNN achieved approximately 0.455. The authors appropriately acknowledge that this level of performance is not yet sufficient for reliable automatic intonation marking and describe the present system as an early-stage prototype.

 The revised manuscript is clearer than the previous version. In particular, the research objectives and research questions are now explicitly stated, the annotation procedure is described in greater detail, and the manuscript provides useful information about the software architecture, preprocessing tools, machine-learning libraries, and hyperparameter exploration. The literature reviewed is generally relevant and reasonably current, particularly in the areas of pronunciation teaching, computer-assisted pronunciation training, Praat, and AI-supported pronunciation analysis.

 Nevertheless, several methodological issues remain and should be addressed before the study can be considered fully scientifically sound.

**1. Model evaluation and data partitioning — must be addressed**

 The manuscript does not clearly describe how the 882 audio clips were partitioned into training, validation, and test sets, or whether cross-validation was used. This information is essential for interpreting the reported classification accuracy. The authors should explicitly report the proportions and numbers of samples used for training, validation, and testing and explain how the partition was generated.

 More importantly, because multiple clips may originate from the same original recording or speaker, the authors should clarify whether the partitioning was performed at the clip, recording, or speaker level. If clips originating from the same recording or speaker appeared in both training and test sets, information leakage could substantially inflate model performance. A recording-level or speaker-level split would therefore be preferable and should be reported explicitly.

**2. Distribution of the 19 intonation classes — must be addressed**

 The manuscript reports a 19-class classification problem but does not provide the frequency or proportion of observations belonging to each class. This information is essential because overall accuracy can be misleading when the class distribution is imbalanced. The authors should add a table showing the number and percentage of examples for each intonation class in the full dataset and, preferably, within the training and evaluation subsets.

**3. Evaluation metrics — must be addressed**

 Model assessment currently relies predominantly on accuracy. For a 19-class classification problem, accuracy alone is insufficient. The authors should report precision, recall, and F1-score, preferably including macro-averaged and weighted-averaged values. A confusion matrix would also be particularly informative because it would show which intonation classes are systematically confused by the model.

 Where feasible, uncertainty around the reported performance should also be provided, for example through repeated cross-validation, confidence intervals, or variability across validation folds. Without these additional measures, the reported difference between the CNN and classical classifiers should be interpreted cautiously.

**4. Annotation reliability — must be addressed or more clearly justified**

 The revised manuscript explains that three PhD-qualified linguists independently annotated the recordings and subsequently resolved disagreements through discussion. This is useful clarification. However, no formal inter-annotator agreement statistic is reported. Since these annotations constitute the reference labels on which model training and evaluation depend, their reliability is central to the validity of the study.

 If the original independent annotations are still available, the authors should calculate an appropriate inter-annotator agreement measure before reconciliation. If this is no longer possible, this limitation should be stated more prominently, and the implications for the reliability of the reference labels and subsequent model evaluation should be discussed.

**5. Participant and recording characteristics**

 Additional information is required regarding the speakers represented by the original 47 recordings. The manuscript should describe, where applicable, the number of speakers, age range, gender distribution, language background, English proficiency, whether speakers contributed multiple recordings, and the source and recording conditions of the audio material. These characteristics are important for assessing the generalisability of the model.

**6. Data augmentation requires methodological clarification**

 The Discussion states that the CNN was trained using an augmented dataset including pitch shifts, noise overlays, and time-frequency masks. However, sufficient procedural details of this augmentation are not provided in the main Methods section. The authors should specify the augmentation parameters, the number or proportion of augmented samples, whether augmentation was applied only to the training set, and how they ensured that augmented versions of the same recording did not enter the evaluation set.

**7. Data availability and reproducibility — must be addressed**

 The availability of the source code through GitHub and archived software repositories is appreciated and improves transparency. However, the Data Availability Statement currently states that no underlying data are associated with the article. This is difficult to reconcile with a study whose reported results depend on 47 recordings, 882 labelled clips, and manually created annotations.

 The authors should clarify whether the annotated dataset, class labels, train/test assignments, and relevant derived features can be made available. If the original audio cannot be shared because of consent, privacy, copyright, or ethical restrictions, this should be stated explicitly. In that situation, the authors should consider sharing de-identified derived data, annotation tables, class distributions, data-partition indices, or other materials sufficient to reproduce the reported analyses.

**8. Interpretation of model superiority**

 The finding that the CNN achieved approximately 0.455 accuracy compared with approximately 0.373 for the best Random Forest model is potentially useful. However, the wording that this difference “confirms the advantage of a deeper architecture” is stronger than the evidence currently supports. Without a clearly described independent test procedure, uncertainty estimates, or repeated validation, the results demonstrate better performance under the reported experimental conditions rather than establishing a general superiority of deep learning. This statement should therefore be moderated.

**9. Conclusions and educational implications**

 I appreciate that the authors now explicitly acknowledge that the achieved accuracy is insufficient for reliable automatic intonation marking and that the software is an early-stage prototype rather than a production-ready system. This is an important and appropriate qualification.

 However, some broader claims regarding personalised feedback, educational access, employability, social inclusion, and sustainability extend beyond what was empirically tested in the present study. No classroom intervention or learner-outcome evaluation was conducted. These statements should therefore be presented as potential future applications or anticipated benefits rather than demonstrated outcomes of the current experiment.

**Overall assessment**

 This is an interesting interdisciplinary project combining phonetics, language education, speech processing, and machine learning. The revised manuscript is clearer and more transparent than the previous version, and the authors appropriately recognise several limitations of the prototype. Nevertheless, the evaluation methodology remains insufficiently documented for a machine-learning study. In particular, the data-partitioning procedure, class distribution, broader classification metrics, annotation reliability, and data availability require further attention.

 The points concerning **data partitioning, class distribution, evaluation metrics, annotation reliability, and reproducibility are essential and should be addressed to make the study scientifically sound**. With these revisions, the manuscript would provide a substantially stronger and more reproducible account of this promising proof-of-concept system.

Is the work clearly and accurately presented and does it cite the current literature?

Yes

If applicable, is the statistical analysis and its interpretation appropriate?

Partly

Are all the source data underlying the results available to ensure full reproducibility?

No

Is the study design appropriate and is the work technically sound?

Partly

Are the conclusions drawn adequately supported by the results?

Partly

Are sufficient details of methods and analysis provided to allow replication by others?

Partly

Reviewer Expertise:

none

I confirm that I have read this submission and believe that I have an appropriate level of expertise to confirm that it is of an acceptable scientific standard, however I have significant reservations, as outlined above.

# Response

**Published:** 2026-09-28

**Authors:** Tamara Oshchepkova

**Affiliations:** Not Applicable, Kuwait

We sincerely thank the reviewer for the careful evaluation of the revised manuscript and for acknowledging the improvements made in response to the previous review. We appreciate the constructive comments regarding the remaining methodological issues, which have helped us further strengthen the transparency and interpretation of the study.

**1. Model evaluation and data partitioning must be addressed.**

 We have revised the manuscript to describe the data-partitioning procedure explicitly. The 882 clips were split 80:20 using sklearn.model_selection.train_test_split (test_size=0.2, random_state=42), resulting in 705 training and 177 test instances. No separate validation set was held out; consequently, all 4,909 classical and 120 CNN configurations were evaluated on the test partition, and the best-performing configurations were selected based on these results.

 The split was performed at the clip level. We also clarify that the 882 clips originated from ten recordings; the previously reported figure of 47 refers to transcript-level annotations rather than separate recordings. Consequently, clips from each recording were present in both the training and test partitions. Speaker-level splitting was not possible because the recordings contain dialogues involving multiple speakers, and speaker identity was not recorded for individual clips. Recording-level grouping is therefore the finest grouping available.

 To assess potential leakage directly, we additionally performed five-fold recording-disjoint cross-validation. This produced a mean accuracy of 0.3681 (SD = 0.0352), compared with 0.37288 for the original clip-level split, indicating no meaningful inflation in overall accuracy. However, macro F1 decreased from 0.154 to 0.1061. Recording-disjoint evaluation will therefore be adopted as the standard approach in future work.

**2. Distribution of the 19 intonation classes must be addressed.**

 Table 5 has been added, reporting the number and percentage of instances in each class for the full corpus and for the training and test subsets. The original annotation scheme contains 26 categories. Of the 1,163 initially identified segments, 882 remained across 19 categories after excluding segments labelled *ignore* or *noise* and those shorter than 100 ms. The class distribution is highly imbalanced: *plain* accounts for 35.4% of the corpus and *stressed_syllable* for 52.5%, while eight categories contain six instances or fewer.

 Because the train-test split was not stratified, five of the 19 categories are absent from the test partition, and four additional categories are represented by only one test instance. These class-distribution limitations and their implications for the reported performance metrics are now discussed in the Results section.

**3. Evaluation metrics must be addressed.**

 Added as Table 6: per-class precision, recall, and F1 scores, together with macro (0.154) and weighted (0.256) averages. The confusion matrix has been deposited with the analysis materials, and its main pattern is described in the text.

 The additional analysis confirms the reviewer’s concern regarding the limitations of overall accuracy. Against a majority-class baseline accuracy of 0.31638, KNN and SVC each correctly classified 56 of 177 instances and therefore did not improve on majority-class prediction; Random Forest correctly classified only ten additional instances. The confusion matrix further shows that errors were not primarily between related tone categories but reflected a strong tendency to predict the two most frequent classes. For example, *high_fall* (0/19 correct), *low_fall* (0/16), *half_stressed_syllable* (0/13), *sliding_head* (0/9), *low_rise* (0/9), and *high_pre_head* (0/8) were assigned predominantly to *plain* or *stressed_syllable*. Only six of the nineteen categories were predicted by the model, and fourteen had F1 = 0. Uncertainty is additionally reflected in the fold variability reported under Point 1.

**4. Annotation reliability - must be addressed or more clearly justified**

 The independent pre-reconciliation annotations were not retained separately from the agreed annotation set; so, an inter-annotator agreement statistic cannot be calculated retrospectively. The Methods section has been revised to distinguish clearly between two stages of annotation. First, intonation markers were placed on the transcripts independently by three linguists and subsequently reconciled through discussion. Second, the agreed text-level markers were aligned with the acoustic signal to produce the clip-level labels used for model training and evaluation. This second stage was performed by a single annotator.

 We therefore clarify that an inter-annotator agreement statistic, had the original independent annotations been retained, would have characterised agreement at the first, text-level annotation stage only. Because the subsequent acoustic alignment and clip-level labelling were performed by one annotator, inter-annotator agreement cannot be calculated for this stage.

 This limitation is now stated explicitly in the manuscript. The reliability of the reference labels underlying model training and evaluation cannot be quantified retrospectively, and all reported performance estimates should therefore be interpreted in this context. In particular, the achievable model-performance ceiling associated with annotation consistency is unknown. This limitation is especially relevant given the fine-grained 19-category classification scheme. The revised protocol for future data collection retains independent annotations at both stages and will report inter-annotator agreement before reconciliation.

**5. Participant and recording characteristics**

 The Methods section has been expanded to describe the participants and recording characteristics and to clarify that ten of the forty-seven recordings were annotated at clip level and used for modelling. The recordings were produced by members of the research team, all first-language speakers of Russian with formal training in English phonetics. They read dialogue texts while deliberately producing the target intonation contours. Individual speakers contributed to more than one recording, and, because the source texts were dialogues, most recordings involved more than one speaker.

 The dialogue texts were drawn from published teaching materials. The recordings were not made under studio conditions, and noise reduction was subsequently applied as part of preprocessing.

 A corresponding generalisability limitation has also been added. The corpus represents elicited, canonical speech rather than spontaneous speech or naturally occurring learner production. Moreover, the speakers, designers of the annotation scheme, and annotators came from the same small research group. The reported results should therefore not be assumed to generalise to spontaneous speech, learner populations, or recordings produced by independent speakers under different conditions.

**6. Data augmentation requires methodological clarification**

 We were unable to substantiate the full original description of the data-augmentation procedure from the retained experimental records and have therefore revised the Methods rather than report parameters that cannot be verified. The available record supports the use of frequency and time masking of the mel-spectrograms; the previously reported references to pitch shifting and noise overlay have been removed.

 The retained records do not allow us to determine reliably the proportion of augmented samples or to verify whether augmentation was applied exclusively after partitioning and only to the training data. We therefore cannot exclude the possibility that augmentation contributed to data leakage between the training and evaluation partitions. This uncertainty is now explicitly reported as a methodological limitation alongside the clip-level partitioning issue. The augmentation results should consequently be interpreted as exploratory rather than as an independent estimate of model generalisation.

**7. Data availability and reproducibility - must be addressed**

 The requested materials are available through the Zenodo archive. The complete Label Studio export (project-1-at-...json) provides the annotated dataset and class labels, including the source recording, time boundaries, and label for each segment. Class distributions are reported in Table 5. The train/test assignment can be reproduced exactly using the documented filtering procedure, random seed, and split parameters. Derived features can be regenerated using the archived notebooks. As part of the revision, we verified the reproducibility of this workflow and recovered the reported Random Forest accuracy of 0.37288 exactly.

 The source audio files themselves are not redistributed because the recordings consist of readings of dialogue texts taken from third-party copyrighted teaching materials. This restriction is now stated explicitly in the Data Availability section.

**8. Interpretation of model superiority**

 The statement that the results “confirm the advantage of a deeper architecture” has been removed. Both results are now reported relative to the majority-class baseline of 0.31638: Random Forest exceeds this baseline by ten correctly classified instances out of 177, while the CNN exceeds it by approximately twenty-four. The difference is no longer interpreted as evidence of a general superiority of deep learning, but in relation to differences in the information available to the two modelling approaches.

 The previously stated three-percentage-point CNN margin has likewise been reduced to a descriptive observation, and the wording “trained and validated” has been corrected because no separate validation set was used.

 The Discussion now provides a likely methodological explanation for the observed difference. The classical features were averaged across the time axis and did not include F0; consequently, they did not directly represent pitch trajectory, although pitch movement is central to the distinction among the target intonation categories. The CNN result is nevertheless interpreted cautiously in light of the data-partitioning and augmentation limitations described above.

**9. Conclusions and educational implications**

 We thank the reviewer for acknowledging the revised prototype framing. In response to this comment, we have revised the broader claims regarding personalised feedback, educational access, employability, social inclusion, and sustainability so that they are presented as potential future applications or anticipated benefits contingent on further validation, rather than as outcomes demonstrated by the present study. We now state explicitly that no classroom intervention was conducted and no learner outcomes were measured. The prototype framing has also been strengthened in the Abstract, Research Context and Objectives, and throughout the Discussion.

# Reviewer report: Reviewer response for version 2

**DOI:** 10.5256/f1000research.205064.r500196

**Published:** 2026-07-20

**Authors:** Dony Marzuki

**Affiliations:** 1 Politeknik Negeri Padang, Padang, Indonesia

This manuscript has been revised substantially but not all major concerns have been addressed. The research objectives as well as research questions have been clearer, and the annotation details have also been explained.  However, the annotation reliability is still not reported, and participants’ demographic info is still not available.  The manuscript does not report how the 19 intonations classes are distributed across the dataset as requested. In addition, the precision, recall, and F1-score asked are still unavailable. instead, the author/s mention that this info is planned for future work, which is not enough. Regarding the dataset availability, the authors now provide GitHub repositories and archived software, but the Data Availability Statement still says: "No underlying data are associated with this article." Hence, the annotated dataset itself remains unavailable. This hindrance will also affect the replicability of the study.   

 New Recommendation: **Approve with Minor Revision**

 It is recommended that the authors address the following points:

1. Describe the train/validation/test partitioning (or cross-validation) used for model evaluation.
2. Report participant characteristics and the distribution of the 19 intonation classes.
3. Include additional evaluation metrics (precision, recall, F1-score).
4. Clarify whether the annotated dataset can be released or explain explicitly why it cannot be shared.
5. Emphasize throughout the manuscript that the reported results represent an early state prototype rather than a validated production-ready system.

Is the work clearly and accurately presented and does it cite the current literature?

Partly

If applicable, is the statistical analysis and its interpretation appropriate?

Partly

Are all the source data underlying the results available to ensure full reproducibility?

No

Is the study design appropriate and is the work technically sound?

Partly

Are the conclusions drawn adequately supported by the results?

Yes

Are sufficient details of methods and analysis provided to allow replication by others?

Partly

Reviewer Expertise:

EFL/ESL instruction, speech fluency, strategy training, autonomous learning

I confirm that I have read this submission and believe that I have an appropriate level of expertise to confirm that it is of an acceptable scientific standard, however I have significant reservations, as outlined above.

# Response

**Published:** 2026-09-14

**Authors:** Tamara Oshchepkova

**Affiliations:** Not Applicable, Kuwait

We sincerely thank the reviewer for the careful reassessment of our revised manuscript and for identifying the concerns that remained insufficiently addressed. The comments have been particularly valuable in helping us recognise where greater methodological transparency and clearer reporting are required. In response, we have substantially revised the relevant sections of the manuscript, providing additional details on the dataset, annotation procedure, evaluation design,performance reporting, and data availability, while explicitly acknowledging limitations where the requested information cannot be recovered retrospectively. A detailed response to each concern and the corresponding revisions are provided below.

**1. Describe the train/validation/test partitioning (or cross-validation) used for model evaluation.**

 We have revised the manuscript to provide the full details of the data partitioning and to explicitly acknowledge its limitations. The 882 labelled clips were divided into training and test sets using an 80:20 split, resulting in 705 training and 177 test instances. No separate validation set or cross-validation procedure was used. The split was performed at the clip level and was not stratified by intonation class. We recognise that this procedure does not provide an independent estimate of generalisation performance. The manuscript has therefore been revised to describe these limitations explicitly and to interpret the reported accuracy values as preliminary results of the prototype-stage evaluation rather than as validated estimates of model performance. Future evaluation will employ recording-disjoint partitioning, a separate validation procedure, and class-stratified evaluation where appropriate.

**2. Report participant characteristics and the distribution of the 19 intonation classes.**

 The recordings were produced primarily by members of the research team reading texts with the target intonation patterns. No external participants were recruited, and demographic information about the speakers was not systematically collected. This has now been clarified in the revised manuscript and acknowledged as a limitation.

 The distribution of the 19 intonation classes has now been determined from the annotation data and is reported in the revised manuscript. The class imbalance and its implications for interpreting overall accuracy are also acknowledged.

**3. Include additional evaluation metrics (precision, recall, F1-score).**

 Precision, recall, and F1-score were not calculated during the original prototype evaluation and cannot be reliably reconstructed from the retained results without rerunning the models. We have therefore explicitly acknowledged the absence of these metrics as a limitation in the revised manuscript. We have also clarified that overall accuracy alone provides an incomplete assessment of performance for a 19-class problem with an uneven class distribution and have moderated our interpretation of the reported results accordingly.

**4. Clarify whether the annotated dataset can be released or explain explicitly why it cannot be shared.**

 We have revised the Data Availability Statement and made the annotated and derived dataset available through the repository on Zenodo. The archive includes the complete Label Studio annotation export, the derived acoustic feature dataset, the annotation scheme, and the Jupyter analysis notebooks. The source audio recordings cannot be redistributed because they consist of readings of dialogue texts derived from third-party copyrighted teaching materials. The revised manuscript now clearly distinguishes between the materials that are openly available and the source audio that cannot be shared for copyright reasons.

**5. Emphasize throughout the manuscript that the reported results represent an early state prototype rather than a validated production-ready system.**

 We have revised the manuscript throughout to consistently present the system as an early-stage proof-of-concept prototype rather than a validated or production-ready tool. Claims regarding model performance, reliability, generalisability, and potential educational applications have been substantially moderated. We have also expanded the discussion of the methodological limitations and explicitly clarified that the reported results should be interpreted as preliminary indicators of prototype performance rather than as evidence of validated system effectiveness.

# Reviewer report: Reviewer response for version 1

**DOI:** 10.5256/f1000research.197356.r488319

**Published:** 2026-06-12

**Authors:** Abdul Hameed Panhwar

**Affiliations:** 1 University of Sindh, Hyderabad, Pakistan

The paper appears to be well organised and potentially research-oriented. However, at some places, more evidence/references are required. For example, paragraph 2 in Section

 'Introduction' has no evidence/references for claims. I would suggest authors should go through and substantiate paper with references/evidence where require.

 The paper also lacks clear stated objectives and research questions or hypotheses. I would suggest that the authors should include and build the study on these.

 Moreover, since I am not a qualified statistician, I cannot competently confirm reliability of data. Therefore, suggest that statistical analysis and findings be rechecked through a qualified statistician.

Is the work clearly and accurately presented and does it cite the current literature?

Yes

If applicable, is the statistical analysis and its interpretation appropriate?

I cannot comment. A qualified statistician is required.

Are all the source data underlying the results available to ensure full reproducibility?

Yes

Is the study design appropriate and is the work technically sound?

Yes

Are the conclusions drawn adequately supported by the results?

Yes

Are sufficient details of methods and analysis provided to allow replication by others?

Yes

Reviewer Expertise:

TESOL, Action Research, collaborative learning

I confirm that I have read this submission and believe that I have an appropriate level of expertise to confirm that it is of an acceptable scientific standard, however I have significant reservations, as outlined above.

# Response

**Published:** 2026-06-27

**Authors:** Tamara Oshchepkova

**Affiliations:** Not Applicable, Kuwait

We sincerely thank the reviewer for the careful evaluation of our manuscript and for the constructive suggestions. We appreciate the recognition of the paper's organisation and research potential. The comments have helped us improve the clarity and methodological presentation of the study. Below, we provide a detailed response to each point and describe the corresponding revisions made to the manuscript.

**At some places, more evidence/references are required**

 We carefully reviewed the manuscript to determine whether additional references were necessary. We believe that the current reference list adequately covers the key and most influential publications relevant to the objectives and scope of this study. While we appreciate the recommendation, we have chosen not to substantially expand the bibliography in order to keep the manuscript concise and focused.

**The paper also lacks clear stated objectives and research questions or hypotheses. I would suggest that the authors should include and build the study on these.**

 The manuscript has been revised to explicitly state the objectives of the study and the research questions guiding the development and evaluation of the proposed prototype. We believe that these additions improve the clarity.

**Suggest that statistical analysis and findings be rechecked through a qualified statistician.**

 We acknowledge the importance of rigorous statistical evaluation for validating machine-learning systems. However, the present manuscript focuses on the development of a proof-of-concept prototype rather than its comprehensive quantitative validation. We believe the analyses reported in the paper are appropriate for the current stage of the project. More advanced statistical evaluation will be performed during the subsequent validation phase using an expanded annotated dataset.

# Reviewer report: Reviewer response for version 1

**DOI:** 10.5256/f1000research.197356.r488321

**Published:** 2026-06-03

**Authors:** Dony Marzuki

**Affiliations:** 1 Politeknik Negeri Padang, Padang, Indonesia

The paper describes a prototype machine-learning system for automatic prosody recognition as a tool for advanced phonetics teaching. The paper is relevant, organized and cites up-to-date research, however its scientific quality is compromised by some methodological problems. The datasets are relatively small for a 19-class intonation classification task. It only has 47 recordings and 882 clips without key information on participant characteristics, class distribution, train-test splits and validation procedures. Annotations reliability is not reported. Statistical analysis is largely based on accuracy without precision, recall, F1-scores and significance tests. Furthermore, the inability to access the underlying dataset and annotations prevents full replication. For an article to be published, the dataset has to be fully documented, measures of annotation reliability, comprehensive evaluation metrics, robust validation processes and public access to the underlying data should be provided.

Is the work clearly and accurately presented and does it cite the current literature?

Partly

If applicable, is the statistical analysis and its interpretation appropriate?

Partly

Are all the source data underlying the results available to ensure full reproducibility?

No

Is the study design appropriate and is the work technically sound?

Partly

Are the conclusions drawn adequately supported by the results?

Yes

Are sufficient details of methods and analysis provided to allow replication by others?

Partly

Reviewer Expertise:

EFL/ESL instruction, speech fluency, strategy training, autonomous learning

I confirm that I have read this submission and believe that I have an appropriate level of expertise to confirm that it is of an acceptable scientific standard, however I have significant reservations, as outlined above.

# Response

**Published:** 2026-06-27

**Authors:** Tamara Oshchepkova

**Affiliations:** Not Applicable, Kuwait

We sincerely thank the reviewer for the careful evaluation of our manuscript and for the constructive comments and suggestions. We appreciate the recognition of the relevance and organisation of the study. The review has helped us improve the manuscript by explicitly acknowledging the current limitations of the proposed prototype. A detailed, point-by-point response to each comment is provided below.

**The datasets are relatively small.**

 The present paper reports the first phase of the project, during which a prototype was developed. As a proof-of-concept study, the prototype was trained and tested on a relatively limited dataset. Following the identification and resolution of the shortcomings observed during the initial experiment, the next phase of the project will focus on verification and validation using a substantially larger dataset. The prototype will be further trained and evaluated on a considerably expanded collection of audio clips to improve its performance and generalisability.

**Annotations reliability is not reported.**

 If the reviewer refers to the manual annotations created during the preprocessing stage, these were independently prepared by three linguists holding PhDs in Applied Linguistics using the classical system of intonational analysis developed by the phoneticians cited in the manuscript. The final reference annotations were established through expert review and discussion. However, formal inter-annotator agreement statistics were not calculated at this preliminary stage of the project. We acknowledge that reporting annotation reliability would strengthen the methodological rigour of the study and have identified this as an important direction for future work.

 If, however, the comment refers to the machine-generated annotations, as noted above, the primary objective of the present study was to develop and describe a prototype rather than to provide a comprehensive evaluation of its performance. Quantitative validation, including the assessment of precision and other performance metrics, is planned for the next stage of the project, as the current prototype does not yet achieve the level of performance required for reliable automatic intonation annotation. The use of a substantially larger annotated corpus is expected to improve the robustness of the model and provide a stronger basis for comprehensive statistical evaluation. This limitation has been acknowledged in the Discussion section of the revised manuscript.

**Statistical analysis is largely based on accuracy without precision, recall, F1-scores and significance tests.**

 The current manuscript focuses primarily on the design and implementation of the prototype, while quantitative evaluation was beyond its original scope. Although a manually annotated reference corpus was used during the initial stage of model development, comprehensive performance evaluation using accuracy, precision, recall, and F1-score has not yet been completed. We acknowledge that such evaluation would substantially strengthen the study and have added this as a limitation and a priority for future work.

**The inability to access the underlying dataset and annotations prevents full replication.**

 The underlying datasets and annotations are publicly available and can be accessed via the links provided in the Declarations section of the manuscript. We have verified that these links are functional and provide unrestricted access to the project materials, which are distributed under the MIT License. We hope this addresses the concern regarding the reproducibility of the study. As stated in the manuscript, the present paper describes the current stage of development of the prototype rather than a fully completed software product.
